# Previous traumatic brain injury is associated with an increased odds for gestational diabetes: a nationwide register-based cohort study in finland

**DOI:** 10.1007/s00592-023-02129-5

**Published:** 2023-06-28

**Authors:** Matias Vaajala, Ilari Kuitunen, Rasmus Liukkonen, Ville Ponkilainen, Maiju Kekki, Ville M. Mattila

**Affiliations:** 1 Faculty of Medicine and Life Sciences, University of Tampere, Tampere, Finland; 2 Department of Pediatrics, Mikkeli Central Hospital, Mikkeli, Finland; 3 Institute of Clinical Medicine and Department of Pediatrics, University of Eastern Finland, Kuopio, Finland; 4 Department of Surgery, Central Finland Central Hospital Nova, Jyvaskyla, Finland; 5 Department of Obstetrics and Gynecology, Tampere University Hospital, Tampere, Finland; 6 Center for Child, Adolescent and Maternal Health Research, Faculty of Medicine and Health Technology, Tampere University, Tampere, Finland; 7 Department of Orthopaedics and Traumatology, Tampere University Hospital, Tampere, Finland

**Keywords:** Gestational diabetes, Traumatic brain injury, Concussion, Epidemiology

## Abstract

**Aims:**

Despite recent findings that traumatic brain injury (TBI) is a possible risk factor for type 2 diabetes (DM2) and that a strong association exists between gestational diabetes (GDM) and the risk for the development of DM2, no previous studies have investigated the effects of TBI on the risk for the development of GDM. Therefore, this study aims to determine the possible association between a previous traumatic brain injury and later gestational diabetes.

**Methods:**

In this retrospective register-based cohort study, data from the National Medical Birth Register were combined with data from the Care Register for Health Care. Women who had sustained a TBI before pregnancy were included in the patient group. Women who had sustained previous fractures of the upper extremity, pelvis, or lower extremity were included in the control group. A logistic regression model was used to assess the risk for the development of GDM during pregnancy. Adjusted odds ratios (aOR) with 95% confidence intervals between the groups were compared. The model was adjusted by prepregnancy body mass index (BMI) and maternal age during pregnancy, the use of in vitro fertilization (IVF), maternal smoking status, and multiple pregnancies. The risk for the development of GDM during different periods following the injury (0–3 years, 3–6 years, 6–9 years, and 9+ years) was calculated.

**Results:**

In total, a 75 g 2-h oral glucose tolerance test (OGTT) was performed on 6802 pregnancies of women who had sustained a TBI and on 11 717 pregnancies of women who sustained fractures of the upper extremity, pelvis, or lower extremity. Of these, 1889 (27.8%) pregnancies were diagnosed with GDM in the patient group and 3117 (26.6%) in the control group. The total odds for GDM were higher after TBI compared to the other traumas (aOR 1.14, CI 1.06–1.22). The odds were highest at 9 + years after the injury (aOR 1.22, CI 1.07–1.39).

**Conclusion:**

The total odds for the development of GDM after TBI were higher when compared to the control group. Based on our findings, more research on this topic is warranted. Moreover, a history of TBI should be considered a possible risk factor for the development of GDM.

**Supplementary Information:**

The online version contains supplementary material available at 10.1007/s00592-023-02129-5.

## Aims

Traumatic brain injury (TBI) has become an increasingly important global health problem [1]. In women, TBI can affect the reproductive system, causing disorders in the menstrual cycle and amenorrhea [2, 3]. Moreover, according to a recent nationwide study carried out in the Finnish population, the incidence of TBIs among fertile-aged women has increased dramatically during the last few decades [4]. These findings are, however, contradicted by another recent study that found that fertility was not impaired in women who sustained a TBI [5]. Thus, more research to investigate the effects of TBIs on the future reproductive health of women is warranted.

According to the findings of a recent review, previous TBIs are a potential risk factor for the future development of type 2 diabetes (DM2) [6]. The direct effects of TBIs on the neuroendocrine system, especially on the hypothalamic–pituitary complex, are well studied [7, 8]. It is known that the hypothalamic–pituitary complex and the targets of the hormones released from this structure (e.g., islets of Langerhans and therefore glucose metabolism), are vulnerable to damage and functional disturbances [7, 8]. In addition, gestational diabetes (GDM) is associated with a higher risk for the future development of DM2 [9, 10]. Interestingly, no previous reports on the effects of TBIs on the development of GDM exist in the published literature.

As TBIs are known to negatively affect the neuroendocrine system [11, 12] and are suspected of being a risk factor for the development of DM2 in the older population [9, 10], pregnancies after TBIs might be at a higher risk for the development of GDM. Thus, this study aims to determine the association between previous TBI and the risk for GDM using nationwide high-quality registers.

## Materials and methods

In this nationwide retrospective register-based cohort study, data from the National Medical Birth Register (MBR) were combined with data from the Care Register for Health Care. Both registers are maintained by the Finnish Institute for Health and Welfare. Data from the registers were combined using the pseudonymized identification numbers of the mothers. The whole study period in our data was from 1 January 1998 to 31 December 2018.

The coverage and quality of the Care Register for Health Care are good [13]. Each trauma hospitalization requiring specialized public healthcare between 1998 and 2018 was included. International classification of diseases 10th revision (ICD-10) codes were used to identify trauma patients. Women who sustained a TBI before pregnancy were included in the patient group. Women with prior fractures of the upper extremity, pelvis, or lower extremity were included in the control group. These women were selected as the reference group as we anticipated them to have a similar background and risk-taking behavior as the women in the major trauma groups, as opposed to women in the general population without any injuries. Fractures of the spine were excluded, as spinal cord injury can be present in such cases. Moreover, as spinal cord injury is known to negatively affect fertility and reproductive system functions, the injury can also affect the outcome of pregnancy [14]. The specific ICD-10 codes with definitions for each trauma included in this study are presented in Supplementary Table 1.

**Fig. 1:**
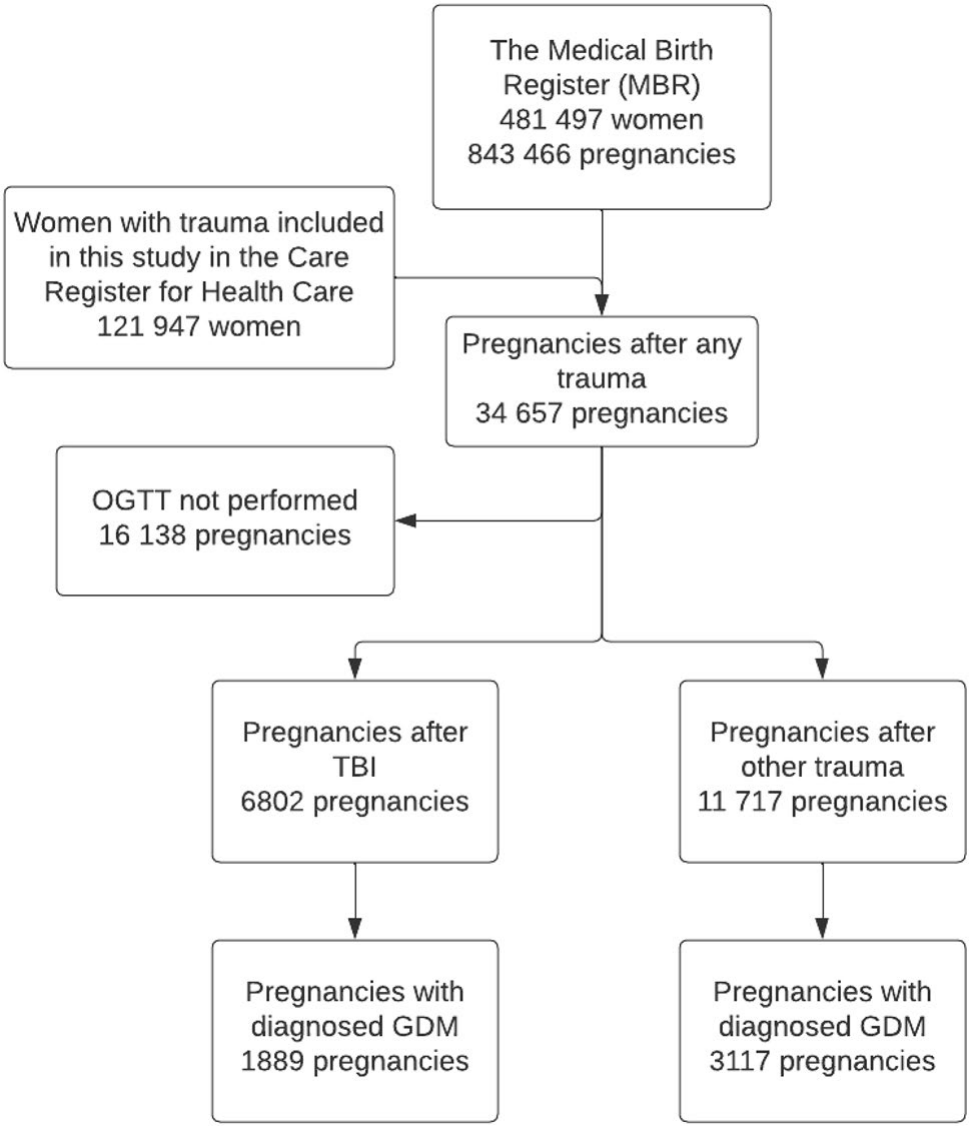
Flowchart of the study population. The risk for gestational diabetes (GDM) was compared between groups. Pregnancies occurring after a traumatic brain injury (TBI) were compared to pregnancies occurring after fractures of the upper extremity, pelvis, or lower extremity. Pregnancies without 75 g 2-h oral glucose tolerance test (OGTT) were excluded from the analysis

The MBR contains information on pregnancies, delivery statistics, and perinatal outcomes of all births with a birthweight of ≥ 500 g or a gestational age of ≥ 22^+0^ and includes information on GDM. GDM was diagnosed using the 75 g 2-h oral glucose tolerance test (OGTT). The MBR has high coverage and quality (the current coverage is nearly 100%) [15, 16]. We included all pregnancies occurring between 2004 and 2018 after one of the traumas included in this study and with OGTT performed during pregnancy. The year 2004 was chosen as the first year for the included pregnancies, as the coding of OGTT started in that year and no information on OGTTs prior to this date is available. Pregnancies without OGTT (*n* = 16,138) were excluded from the analysis. The process used to form the study groups is shown as a flowchart in Fig. 1.

## Statistics

Continuous variables were interpreted as mean with standard deviation or as median with interquartile range based on the distribution of the data. Categorized variables were presented as absolute numbers and percentages. Student’s *t*-test, Mann–Whitney *U*-test, and Chi-squared tests were used for group comparisons. A *p* value under 0.05 was considered statistically significant. A logistic regression model was used to assess the primary outcomes. The exposure variable was the type of trauma, and the primary outcome was the diagnosed GDM. Adjusted odds ratios (aORs) with 95% confidence intervals (CIs) between the groups were compared. The model was adjusted by maternal age during pregnancy, maternal BMI before and at the beginning of pregnancy, the use of in vitro fertilization (IVF), maternal smoking status (yes/no), and multiple pregnancies (yes/no). Based on the previous literature, all the above variables are known to be risk factors for GDM [17–20]. As there is a scarcity of information on the effects of TBIs on the later development of GDM, the extent to which the adverse effects of TBIs impact glucose metabolism and whether the effects occur with a delay, we also tested the risk for the development of GDM in different time periods following the injury (0–3 years, 3–6 years, 6–9 years, and 9+ years). The results of this study are reported using STROBE guidelines [21]. Statistical analysis was performed using R version 4.0.3 (R Foundation for Statistical Computing, Vienna, Austria).

## Ethics

Both the National Medical Birth Register (MBR) and the Care Register for Health Care had the same unique pseudonymized identification number for each patient. The pseudonymization was done by the Finnish data authority Findata. The authors did not have access to the pseudonymization key, as it is maintained by Findata. In accordance with Finnish regulations, no written informed consent was required because of the retrospective register-based study design, and the patients were not contacted. Permission for the use of the data was granted by Findata after the evaluation of the study protocol. (Permission number: THL/1756/14.02.00/2020).

## Results

In total, OGTT was performed on 6802 pregnancies of women in the patient group and on 11 717 pregnancies of women in the control group. Of these, 1889 (27.8%) pregnancies were diagnosed with GDM in the patient group and 3117 (26.6%) in the control group. When compared to the patient group, maternal age during pregnancy was higher in the control group (mean 31.2 years vs. 29.6 years, *p* > 0.001). A notably higher rate of women were smokers during pregnancy in the patient group when compared to the control group (25.6% vs. 19.0%, *p* > 0.001). (Table 1) The total odds for the development of GDM were higher after TBI when compared to other traumas (aOR 1.14, CI 1.06–1.22). The odds were highest at 9 or more years after the injury (aOR 1.22, CI 1.07–1.39) (Table 2).

**Table 1:** Background information on the study groups

Total number	TBI group		Control group	
	6802		11,717	
	*n*	%	*n*	%
Age at the time of trauma (mean; sd)	22.6 (5.6)		23.7 (6.0)	
Age at the time of pregnancy (mean; sd)	29.6 (5.4)		31.2 (5.2)	
Maternal BMI (mean; sd)	26.7 (5.5)		26.8 (5.6)	
BMI unknown	73	1.1	156	1.3
*Maternal smoking status*				
Confirmed smoker*	1742	25.6	2230	19.0
Unknown	155	2.3	250	2.1
Use of in vitro fertilization	22	0.3	38	0.3
Multiple pregnancies	101	1.5	184	1.6
Pregnancies with gestational diabetes	1889	27.8	3117	26.6

*Smoker only during 1st trimester or smoker also during later trimesters

**Table 2:** Time-stratified and overall time-adjusted odds ratios (aOR) with 95% confidence intervals (CIs) for the event of the development of gestational diabetes after trauma

	aOR* (CI)
Total odds	1.14 (1.06–1.22)
Risk at 0–3 years after injury	1.03 (0.90–1.19)
Risk at 3–6 years after injury	1.15 (1.00–1.33)
Risk at 6–9 years after injury	1.10 (0.63–1.28)
Risk at 9 or more years after injury	1.22 (1.07–1.39)

Mothers who suffered traumatic brain injuries (TBIs) were compared to those who suffered fractures of the upper extremity, pelvis, or lower extremity

*Adjusted by maternal age and BMI during pregnancy, use of in vitro fertilization, maternal smoking status, and multiple pregnancies

## Discussion

The main finding of the present study is that the total odds for GDM were higher after TBI. The odds for GDM were also higher in the patient group 9 or more years after the trauma. To the best of our knowledge, no previous studies have examined the effects of TBI on the risk of the development of GDM in later pregnancies. The results of this study will, therefore, provide baseline information on the risk of GDM in later pregnancies associated with TBI.

Recently, a review hypothesized that a history of TBI might be a possible risk factor for the development of DM2 [6]. However, as the higher risk was observed in older patients who had had a previous TBI, DM2 might have occurred a long time after the injury was sustained [6]. Interestingly, our results show that the risk for GDM was highest 9 or more years after the initial injury. The exact reason for this finding remains unclear, but there are two possible explanations for this increase after such a long period of time. First, the effects of TBIs on the risk for the development of GDM might come with a delay and are not, therefore, observed in pregnancies that occur immediately after the injury is sustained. This might be supported by the previous literature, as TBIs are also known to cause complications after a long period of time has passed [22]. Another possible explanation might be that the effects of TBIs on the risk for the development of GDM are more likely to show up in the pregnancies of older women. Due to the nature of our data (we only have information on injuries sustained by females aged 15–49 years), those pregnancies occurring after 9 or more years involve women of higher maternal age. According to previous studies, the secretory patterns of hormones produced by the hypothalamic–pituitary axis change during aging. A similar change is also observed in the sensitivity of the axis to negative feedback by end hormones [23]. Additionally, glucose homeostasis tends toward disequilibrium with increasing age [23]. This could mean that women with a history of TBIs and vulnerability to disequilibrium of the endocrine system due to aging might be prone to the development of GDM.

Also, the psychological aspects of TBIs should be taken into account when considering the association between TBIs and GDM, even though the psychological mechanisms are still not well understood. E.g., It is known that TBIs are known to be a major risk factor for post-traumatic stress disorder (PTSD) [24], which is found to be associated with DM2 and GDM [25, 26]. Even mild TBIs are known to increase the risk for PTSD [27]. In addition to psychological symptoms, PTSD is known to have an effect on the homeostasis of cortisol and catecholamines [28]. Both catecholamines and cortisol have a strong hyperglycaemic effect, which might have an effect on the development of GDM and DM2, as discussed in the recent review [6]. Additionally, the stress, sleep disturbances, and emotional toll of recovering from a traumatic brain injury can possibly lead to unhealthy lifestyle habits, such as poor diet and lack of exercise, which can further contribute to the development of GDM. However, as our data includes only information from the MBR and the TBI diagnoses from the Care Register for Health Care, it does not provide any data on the psychological status of women, such as stress, anxiety, or depression, since these factors are not routinely recorded in the MBR or the Care Register for Health Care. Therefore, further research is needed to explore the complex interplay between TBIs, psychological factors, and gestational diabetes, using a comprehensive approach that includes both medical and psychological data.

TBIs are known to negatively affect the hypothalamic–pituitary complex and thus also the endocrine system [7, 8]. Based on the results of the present study, it appears that possible alterations in the hormonal system might be a risk factor for the development of GDM. However, due to the crude nature of our data, the exact reason for this increased risk remains unknown and further research, using larger datasets and more precise patient information, should be conducted. Moreover, another reason why more research should be conducted in the future is that the incidence of both TBIs and pregnancies with a diagnosis of GDM is increasing [4, 29]. Indeed, future research is important because GDM is known to have many negative effects on the health of the fetus and increases the risk for the development of DM2 [9, 10, 30].

The strength of our study is its use of large nationwide register data with excellent coverage and quality [13, 15, 16]. The register data used in our study are routinely collected using structured forms with national instructions, which ensures good coverage and reduces possible reporting and selection biases. However, the present study has several limitations that should be addressed. The main limitation of our study is that a relatively high proportion of pregnancies were excluded from the analysis because OGTT was not performed. After 2008, the screening methods for GDM were changed to comprehensive screening. This meant that the testing rates for GDM increased notably toward the end of the study period [31]. A further limitation is the missing clinical information on the traumas included in this study (e.g., radiological findings, trauma mechanisms, and cause of trauma). As a result, the severity of the TBIs included in the study remains unknown. Another limitation of our study is the missing clinical information on whether patients had a history of either type I or type II diabetes. This limitation should, therefore, be considered when interpreting the results of this study. However, as our study sample was large, we believe that any possible bias did not markedly affect our results.

## Conclusion

The total odds for the development of GDM after TBI were higher when compared to the control group. In particular, a previous TBI and increasing age might be possible risk factors for the development of GDM. Therefore, a history of TBIs should be acknowledged as a possible risk factor for the development of GDM, and the results gained from this study should be used in the prevention and screening of GDM.

Based on our findings, further research is warranted and a history of TBI should be acknowledged as a possible risk factor for the development of GDM.

## Supplementary Information

Below is the link to the electronic supplementary material.

Supplementary file1 (PDF 44 KB)
