# Supplementary material for: Previous traumatic brain injury is associated with an increased odds for gestational diabetes: a nationwide register-based cohort study in finland
Source: Acta Diabetol. 2023 Jun 28;60(10):1399–404. doi: 10.1007/s00592-023-02129-5 (PMC10442252; doi:10.1007/s00592-023-02129-5)
Supplement: Supplementary file 1 — Supplementary file1 (PDF 44 KB) [file 592_2023_2129_MOESM1_ESM.pdf]

Supplementary Table 1: ICD-10 codes with definitions for each trauma included in this study. Traumatic brain injuries were compared to a control group, which consisted of fractures of the upper extremity, pelvis or lower extremity

Traumatic brain injuries	Patient group
ICD-10 code	Definition
S06.0	Concussion
S06.1	Traumatic cerebral oedema
S06.2	Diffuse traumatic brain injury
S06.3	Focal traumatic brain injury
S06.4	Epidural haemorrhage
S06.5	Traumatic subdural haemorrhage
S06.6	Traumatic subarachnoid haemorrhage
S06.8	Other specified intracranial injuries
S06.9	Unspecified intracranial injury
Fractures of upper extremity, pelvis, or lower extremity	Control group
ICD-10 code	Definition
S52.0	Fracture of upper end of ulna
S52.1	Fracture of upper end of radius
S52.2	Fracture of shaft of ulna
S52.3	Fracture of shaft of radius
S52.5	Fracture of lower end of radius
S52.6	Fracture of lower end of ulna
S52.9	Unspecified fracture of forearm
S62.0	Fracture of navicular bone of wrist
S62.1	Fracture of other and unspecified carpal bone
S62.2	Fracture of first metacarpal bone
S62.3	Fracture of other and unspecified metacarpal bone
S62.4	Multiple fractures of metacarpi
S42.0	Fracture of clavicle
S42.1	Fracture of scapula
S42.2	Fracture of upper end of humerus
S42.3	Fracture of shaft of humerus
S42.4	Fracture of lower end of humerus
S42.9	Fracture of shoulder girdle, part unspecified
S32.1	Fracture of sacrum
S32.3	Fracture of ilium
S32.4	Fracture of acetabulum
S32.5	Fracture of pubis
S32.7	Multiple fractures of lumbar spine and pelvis
S32.8	Fracture of other parts of pelvis
S32.9	Fracture of unspecified parts of lumbosacral spine and pelvis
S72.0	Fracture of head and neck of femur
S72.1	Pertrochanteric fracture
S72.3	Fracture of shaft of femur
S72.4	Fracture of lower end of femur

S72.7	Multiple fractures of femur
S72.8	Other fracture of femur
S72.9	Unspecified fracture of femur
S82.0	Fracture of patella
S82.1	Fracture of upper end of tibia
S82.2	Fracture of shaft of tibia
S82.3	Fracture of lower end of tibia
S82.4	Fracture of shaft of fibula
S82.5	Fracture of medial malleolus
S82.6	Fracture of lateral malleolus
S82.8	Other fractures of lower leg
S82.9	Unspecified fracture of lower leg
S92.0	Fracture of calcaneus
S92.1	Fracture of talus
